# 肿瘤相关成纤维细胞对于免疫细胞的调节作用研究现状

**DOI:** 10.3779/j.issn.1009-3419.2022.101.04

**Published:** 2022-03-20

**Authors:** 广 慕, 文豪 张, 晶晶 黄, 志鹏 陈, 俊 王

**Affiliations:** 210000 南京，南京医科大学附属第一医院胸外科 Department of Thoracic Surgery, The First Clinical Medical College of Nanjing Medical University, Nanjing 210000, China

**Keywords:** 肿瘤微环境, 肿瘤相关成纤维细胞, 肿瘤浸润型免疫细胞, 免疫调节, 肿瘤免疫, Tumor microenvironment, Tumor-associated fibroblast, Tumor-infiltrating immune cells, Immunoregulation, Tumor immunity

## Abstract

肿瘤相关成纤维细胞（cancer-associated fibroblasts, CAFs）和肿瘤浸润性免疫细胞是肿瘤微环境（tumor microenvironment, TME）中重要的组成成分。二者在肿瘤微环境中相互通信，在肿瘤的发生和发展中发挥着重要作用。CAFs具有很大的异质性，不同的CAFs亚群存在着不同的功能。同时其又可以通过分泌多种细胞因子、趋化因子等方式实现对肿瘤浸润性免疫细胞的调节，从而进一步影响肿瘤的发生、发展、侵袭、转移等生物学行为。本文对国内外近年来有关CAFs在TME中对浸润性免疫细胞调节作用的研究现状作一综述。

## Abstract (en)

Cancer-associated fibroblasts (CAFs) and tumor-infiltrating immune cells are the most essential components of the tumor microenvironment (TME). They communicate with each other in tumor microenvironment and play a critical role in tumorigenesis and development. CAFs are very heterogeneous and different subtypes of CAFs display different functions. At the same time, it can contribute to the regulation of the function of tumor-infiltrating immune cells and eventually result in the carcinogenesis, tumor progression, invasion, metastasis and other biological behaviors of tumors by producting various growth factors and cytokines *etc*. Based on the current research results at home and abroad, this paper reviews the recent research progress on the regulation of CAFs on infiltrating immune cells in tumor microenvironment.

已有的研究^[1]^发现，肿瘤的发生发展不仅取决于肿瘤细胞本身，还与肿瘤细胞所处的环境即肿瘤微环境（tumor microenvironment, TME）密切相关。TME主要由血管、肿瘤相关成纤维细胞（cancer-associated fibroblasts, CAFs）、细胞外基质（extracellular matrix, ECM）和浸润性免疫细胞组成^[2]^。其中，CAFs是一类异质群体，是肿瘤细胞外最主要的基质成分，可以分泌多种细胞因子对肿瘤细胞的发生发展进行调控^[3]^。微环境中的免疫细胞主要包括肿瘤浸润性淋巴细胞（tumor infiltrating lymphocytes, TILs）、肿瘤相关巨噬细胞（tumor-associated macrophages, TAM）、树突状细胞（dendritic cells, DCs）、骨髓来源的抑制性细胞（myeloid-derived suppressor cells, MDSCs）和自然杀伤细胞（natural killer cell, NK）等，它们对于肿瘤的生物学行为同样有着重要的调控作用。本文主要总结了近年来关于CAFs对于微环境中免疫细胞的调节作用的研究成果，强调了它们在肿瘤进展和免疫逃逸中的协同作用，以期发现影响肿瘤进展的新型分子靶点和通路。

## 1 CAFs的起源和异质性

成纤维细胞是间质中最丰富的细胞类型之一^[4]^。我们把TME中被激活的成纤维细胞称之为CAFs^[5]^。尽管对于CAFs已经有大量的研究，但是有关其起源的多重性，目前仍在讨论中。组织驻留成纤维细胞是CAFs的主要来源之一^[6, 7]^。肿瘤细胞分泌的转化生长因子-β（transforming growth factor-β, TGF-β）、血小板来源的生长因子（platelet derived growth factor, PDGF）和成纤维细胞生长因子2（fibroblast growth factor 2, FGF-2）、基质衍生因子-1（stromal cell-derived factor 1, SDF-1）等可以激活组织成纤维细胞^[8-10]^。当然，由于肿瘤种类的不同，其分泌的调控因子也不相同。有研究^[11, 12]^表明，静止状态的胰腺星状细胞（pancreatic stellate cells, PSCs）和肝星状细胞（hepatic stellate cells, HSCs）在TGF-β和PDGF的激活下表达α-平滑肌肌动蛋白（alpha smooth muscle actin, α-SMA），α-SMA反作用于PSCs和HSCs，将其激活为CAFs。另外，胰岛素样生长因子1（insulin-like growth factor 1, IGF-1）也可以对HSCs有激活作用^[13]^。CAFs还可以来源于骨髓，有研究^[14]^表明，骨髓间充质干细胞（bone marrow mesenchystem cells, BM-MSCs）在TGF-β1的介导下分化为不同的CAFs亚群。髓源性MSCs分化为CAFs后，可以表达α-SMA和成纤维细胞活化蛋白（fibroblast activation protein, FAP）^[15]^。有研究^[16]^发现，TGF-β1可促进增殖的内皮细胞表型转化为成纤维细胞样细胞。TGF-β1能够诱导成纤维细胞特异性蛋白1（fibroblast specific protein-1, FSP1）和SMA等间充质标志物的表达刺激内皮细胞向间充质细胞转变（endothelial-mesenchymal transition, EndMT）。此外，也有研究^[17-25]^表明，脂肪细胞、上皮细胞、周细胞、平滑肌细胞也可以转化为CAFs。CAFs的多重起源理论在某种程度上解释了其异质性的原因。近年来有学者^[26]^在胰腺癌中发现了两个对立的CAFs亚型：肌成纤维细胞性CAFs（myofibroblastic CAFs, myCAFs）和炎性CAFs（inflammatory CAFs, iCAFs）。myCAFs位于癌细胞附近，可以大量表达α-SMA，而iCAFs离肿瘤细胞较远，表达α-SMA较少，但分泌较多的白介素（interleukin, IL）-6和其他炎症因子[如IL-8、IL-11和白血病抑制因子（leukemia inhibitory factor, LIF）]，可能通过刺激STAT3信号通路参与免疫抑制^[27]^。在三阴型乳腺癌（triple negative breast cancer, TNBC）中，根据不同的成纤维细胞标志物，将CAFs分为4个亚群（S1-S4）^[28]^。激活标志物的差异表达主要包括α-SMA、FAP、血小板来源的生长因子受体β（platelet-derived growth factor receptor β, PDGFRβ）、成纤维细胞特异性蛋白-1（fibroblast specific protein-1, FSP-1）、caveolin 1（CAV-1）和CD29。所有CAF亚型均有较低的CAV-1水平。CAF-S1亚群主要表达这6种标志物，其中FAP和α-SMA高表达；CAF-S2亚群表达6种标志物的低水平；CAF-S3亚群α-SMA和FAP均为阴性，但其余4个标志物均为阳性；CAF-S4亚群无FAP，但α-SMA和CD29较高。在定位方面，CAF-S1和CAF-S4主要出现在TNBC肿瘤中，人类表皮生长因子受体2（human epidermalgrowth factor receptor-2, HER2）+ 肿瘤中附加CAF-S4。CAF-S3在HER2+和TNBC肿瘤中具有肿瘤旁定位。最后，CAF-S2既存在于肿瘤区，也存在于肿瘤旁区，主要存在于腔内A亚型^[28]^。CAF-S1亚群刺激Treg细胞的分化、募集和活化，从而促进肿瘤免疫抑制，还可以分泌CXCL12和TGF-β，促进癌细胞迁移^[28, 29]^。CAF-S4亚群则通过NOTCH通路促进肿瘤细胞迁移和侵袭^[30]^。观察表明，微环境中可能存在pCAFs（cancer-promoting CAFs）和rCAFs（cancer-restraining CAFs）两种不同的群体^[31]^。pCAFs主要通过表达FAP-α或α-SMA多种途径抑制抗肿瘤免疫^[32, 33]^，而rCAFs广泛分布于结肠癌、膀胱癌、肠癌等各种肿瘤中^[34-36]^。有研究^[37]^发现，rCAFs可以抑制肿瘤的生长，而Meflin是一种以糖基磷脂酰肌醇为锚定的蛋白，可以作为rCAFs抑制胰腺导管腺癌（pancreatic ductal adenocarcinoma, PDAC）进展的标志。目前，由于缺乏特异性标志物来识别不同的CAFs，这为我们进一步了解CAFs的异质性增添了不少困难。

## 2 CAFs对TILs的调节作用

TILs被认为是对特异性免疫应答具有高度反应性的细胞亚群，主要由CD4^+^ T细胞和CD8^+^ T细胞两大细胞亚群构成^[38]^。CD4^+^ T细胞主要分为Th1细胞、Th2细胞、Th17细胞和Treg细胞^[39]^。活化的Th1细胞释放IL-2、干扰素γ（interferon-γ, IFN-γ）和肿瘤坏死因子α（tumor necrosis factor-α, TNF-α）等细胞因子，通过介导细胞免疫诱导肿瘤细胞的凋亡；而活化的Th2细胞释放IL-4、IL-5、IL-10和IL-13等细胞因子，通过介导体液免疫发挥促进肿瘤生长的作用^[40]^。CAFs在被TNF-α和IL-1β激活后，分泌胸腺基质淋巴生成素（thymic stromal lymphopoietin, TSLP），通过调节骨髓状DCs，促进Th2的极化。在原发性肿瘤中，使用FAP^+^CAFs DNA疫苗可以显著增加IL-2、IL-7 Th1细胞因子的表达，同时还可以显著诱导TME中IL-4、IL-6 Th2细胞因子的减少，从而增加细胞毒性T淋巴细胞（cytotoxic lymphocyte, CTL）的杀伤力^[41, 42]^。Treg细胞是一类调节性T细胞，可以分泌IL-4、IL-10及TGF-β等细胞因子产生免疫抑制，促进肿瘤的发展^[43]^。CAFs在肺癌中表达的环加氧酶2会导致其分泌前列腺素E2，而前列腺素E2可诱导FOXp3的表达，FOXp3是Treg的重要标记，在Treg细胞功能中发挥重要作用^[44]^。有研究^[28]^表明，CAF-S1通过分泌CXCL-12吸引CD4^+^CD25^+^ T淋巴细胞，并被OX40L、程序性细胞死亡蛋白1配体2（programmed cell death 1 ligand 2, PD-L2）和JAM2保留。此外，CAF-S1还可增加T淋巴细胞存活率，并通过B7H3、CD73和DPP4促进其分化为CD25（high）FOXP（high）Treg。有学者^[45]^发现CD73^+^γδTregs是乳腺癌中主要的浸润性T细胞，并且比CD4^+^ T细胞和CD8^+^ T细胞有着更强大的免疫抑制效应。他们进一步发现了CAFs分泌IL-6，并通过IL-6/STAT3通路诱导CD73^+^γδTregs分化以及产生更多的腺苷，从而形成了强大的肿瘤免疫抑制功能。接着，他们又证明了CD73^+^γδTregs可以通过腺苷/A2BR/p38MAPK信号通路反向促进CAFs分泌IL-6，形成IL-6-腺苷正反馈回路。CAFs通过释放IL-1β激活核因子κB（nuclear factor κB, NF-κB）来诱导CCL-22 mRNA的表达，而FOXp3的表达又和CCL-22呈正相关。可见，IL-1β-CCL22-CCR4轴会促进细胞转化和Treg浸润，从而引起肿瘤的免疫抑制^[46]^。在肺癌微环境中，大多数CD8^+^ T细胞经活化后转变为CTL发挥肿瘤杀伤作用^[47]^。有实验^[48]^证明，当CAFs数量较低时，瘤内和瘤周均有大量的CD8^+^ TILs存在；当CAFs数量较多时，尽管瘤周CD8^+^ TIL依然较多，但瘤内的数量显著减少。与之相反，CAFs较多时，瘤内FOXp3^+^ TILs较多。这表明CAFs可能通过调节TIL的迁移，实现免疫抑制。进一步研究显示，CAFs抑制CD8^+^ TIL向瘤内浸润，而促进FOXp3^+^ TIL瘤内浸润。近年来也发现CAFs参与抗原交叉提呈过程，通过PD-L2和Fas配体介导的抗原特异、抗原依赖途径杀伤CD8^+^ T淋巴细胞，从而使肿瘤细胞逃脱免疫系统的攻击。有研究^[49]^发现，在胰腺导管腺癌中存在一类表达主要组织相容性复合体（major histocompatibility complex, MHC）II类和CD74的新CAF亚群——apCAFs（antigen-presenting CAFs），研究证明从原位肿瘤分离的apCAFs具有在共培养的T细胞中显示出诱导CD25和CD69的能力。CAFs利用肿瘤抗原交叉呈递和关键免疫检查点配体的同步上调，驱动抗原特异性T细胞死亡和细胞毒性T细胞的功能损伤，使肿瘤细胞逃避免疫攻击。有学者^[50]^发现，CAFs分泌的TNF-β可以诱导CD8^+^ T细胞表达FOXP3，促进其向CD8^+^ Treg细胞转化。CAFs通过IL-6调节TME中免疫抑制TIL数量。当IL-6的产生被阻断时，可改善已有的肿瘤免疫，提高常规免疫治疗的疗效。

## 3 CAFs对MDSCs的调节作用

MDSCs是一种异质细胞群，可强烈抑制T细胞和NK细胞的抗肿瘤活性并刺激Treg细胞，导致肿瘤进展^[51]^。CAFs可通过SDF-1a/CXCR4途径趋化单核细胞，并通过IL-6介导的STAT3激活诱导单核细胞分化为MDSCs。这些MDSCs以STAT3依赖的方式抑制T细胞增殖，改变T细胞的表型和功能，上调IL-10，下调IFN-γ，诱导Treg分化和T细胞凋亡^[52]^。基于这些发现，未来可以考虑把IL-6和GM-CSF作为肿瘤患者MDSC诱导抑制的治疗靶点。有研究^[53]^表明，CAFs与肿瘤细胞共培养时，会上调CCL7、CXCL1、CXCL2、CXCL8的表达水平，这些趋化因子会进一步促进MDSCs的募集。肿瘤细胞可产生集落刺激因子1（colony-stimulating factor 1, CSF1），CSF1是一种负调控趋化因子，通过CAF将PMN-MDSC募集到肿瘤部位，从而促进免疫抑制^[54]^。也有学者^[55]^发现通过抑制吲哚胺-2, 3-双加氧酶1（indoleamine 2, 3-dioxygenase1, IDO1）和NADPH氧化酶NOX2和NOX4，从而减少CAFs诱导的MDSCs中活性氧（reactive oxygen species, ROS）的产生，进而恢复CD8^+^ T细胞的增殖，清除ROS破坏了CAFs-MDSCs轴，为逆转CAFs介导的免疫抑制微环境提供了潜在的治疗途径。但是如何有效清除微环境中过多的ROS以维持氧化还原平衡，改善CD8^+^ T细胞的功能，值得进一步研究。

## 4 CAFs对TAMs的调节作用

TAMs是NSCLC免疫浸润的重要成分，具有高度可塑性并表现出多种表型，包括M1型（经典激活，抗肿瘤活性的促炎性反应）和M2型（非经典激活，促血管生成和原始肿瘤活性的免疫抑制）^[56]^。有研究^[57-59]^显示，CAFs可能通过细胞因子MCP-1和SDF-1的介导实现对单核细胞的募集。研究人员分别通过阻断MCP-1或SDF-1受体（CXCR4），抑制MCP-1或SDF-1活性，结果显示单核细胞的迁移能力降低。研究发现，虽然SDF-1和MCP-1都与CAF和乳腺癌细胞介导的单核细胞招募相关；SDF-1似乎在CAFs诱导的单核细胞招募中更为重要，而MCP-1在乳腺癌细胞介导的单核细胞迁移中更为突出。该研究团队还证明没有肿瘤细胞的存在，CAFs也能够自行诱导PD-1+ TAM表型，在诱导PD-1表达方面，CAFs与肿瘤细胞一样有效^[57]^。有研究^[60]^发现，肿瘤细胞可以分泌IL-6和GM-CSF，刺激CAFs激活，而激活的CAFs又可以进一步促进单核细胞向M2分化。当使用IL-6抗体和GM-CSF抗体时，可以观察到肿瘤重量的降低，降低了肿瘤的发生，这也进一步印证了其观点。此外，CAFs可诱导IL-6、IL-8、TGF-β、IL-10等募集单核细胞并向M2型巨噬细胞分化。经CAFs培养的M1巨噬细胞M2标志物表达增加，抗炎细胞因子IL-10的产生增加，而促炎症细胞因子IL-12的产生减少；提示CAFs也能诱导M1巨噬细胞向M2巨噬细胞转分化^[60]^。Cohen等^[61]^证实，Chi3L1在从乳腺肿瘤和转基因小鼠肺转移瘤分离的CAFs以及人乳腺癌间质中高度上调。体内成纤维细胞内的*Chi3L1*基因消融可降低肿瘤生长、巨噬细胞募集和向M2样表型的重编程，增强CD8^+^和CD4^+^ T细胞对肿瘤的浸润，并促进Th1表型的转化。同样，M2巨噬细胞与CAFs的关系是相互的，M2巨噬细胞也能够影响成纤维细胞的间充质-间充质转化，导致其反应性增强^[62]^。尽管有大量研究表明，肿瘤细胞CAFs和TAMs之间通过分泌各种细胞因子相互调控，但是由于细胞因子网络的复杂性，具体的机制尚未完全揭示，这为我们的研究提供了方向，也为治疗提供了靶点。

## 5 CAFs对DCs的调节作用

DCs是有效的抗原呈递细胞，能够通过I类和II类MHC复合物、共刺激分子和黏附分子的表达启动初级免疫反应，是启动、调控和维持免疫应答的中心环节，在诱导抗肺癌免疫应答中起重要作用^[63]^。有研究^[64]^表明，色氨酸的代谢产物Kyn是一种重要的微环境因子，可以抑制DCs的分化，诱导癌症生长和迁移。而CAFs可以表达IDO或色氨酸-2, 3-双加氧酶（recombinant tryptophan-2, 3-dioxygenase, TDO）对色氨酸进行分解代谢，产生更多的Kyn，进而抑制DC功能，促进肿瘤免疫。关于CAFs对于色氨酸代谢的影响，还有待于进一步的探究，这也为我们提供了一个新的治疗靶点。有研究^[65]^发现，来源于肝细胞癌（hepatocellular carcinoma, HCC）的CAFs可以促进调节性DC的生成，其特征是共刺激分子的低表达、高抑制性细胞因子的产生和免疫反应的增强调节，包括T细胞增殖障碍和通过IDO上调促进调节性T细胞（Treg）扩增。该研究还发现，当使用STAT3特异性抑制剂时，肝脏CAFs-DC中的IDO生成显著下调，提示肝脏CAFs-DC分泌IDO是由激活的STAT3介导。进一步研究发现，肝细胞癌来源的CAFs能够通过IL-6介导的STAT3激活将正常DC转化为IDO产生细胞，从而形成肿瘤的免疫抑制。有学者^[66]^尝试将DCs与CAFs融合，发现DC/CAF融合细胞激活的T细胞在体外可以产生强烈的CTL反应。DC/CAFs融合比未成熟DCs表达更高水平的共刺激CD80、CD86和MHC II分子，实验研究表明DC/CAF融合细胞免疫可显著降低H22肿瘤的生长并延长BALB/C荷瘤小鼠的存活时间。这些结果表明DC/CAF融合细胞作为一种新型抗肿瘤疫苗具有刺激T细胞的潜力。

## 6 CAFs对肿瘤NK细胞的调节作用

NK细胞通过直接识别并杀伤肿瘤细胞在抗肿瘤免疫中发挥关键作用^[67]^。自然杀伤组2成员D（natural killer group 2 member D receptor, NKG2D）是NK细胞的激活受体之一，对NK细胞的激活至关重要。NKG2D的两个配体MICA/B可以在肿瘤细胞表面表达。有研究^[68]^显示，黑色素瘤微环境中CAF增加基质金属蛋白酶（matrix metalloproteinase, MMPs）的分泌可降低MICA/B的表达，从而进一步降低NK细胞对依赖NKG2D的黑色素瘤癌细胞的细胞毒性活性。CAFs还可通过分泌前列腺素E2（prostaglandin E2, PGE2）和/或IDO降低NK细胞表面几种NK激活受体（包括NKp30、NKp44和NKG2D）的表达，从而降低NK细胞对肿瘤靶细胞的杀伤活性^[69]^。CAFs的细胞表面可以表达脊髓灰质炎病毒受体（poliovirus receptor, PVR），PVR是NK激活受体DNAX辅助分子-1（DNAM-1）的重要配体，流式细胞术发现相对于正常成纤维细胞，PVR在CAFs细胞表面表达降低，使用抗PVR的siRNA（PVRsi）来下调NEF中PVR的表达，与PVRsi转染的正常成纤维细胞共培养的NK细胞杀伤活性下降到对照转染正常成纤维细胞的大约1/3。PVR表达的降低和对NK细胞活性的影响与CAFs大致相当。这些数据提示PVR在CAFs中的表达降低是CAFs诱导的NK细胞活性抑制的关键。CAFs可产生TGF-β从而抑制NK细胞IFN-γ的表达，进而阻碍Th1的分化和抑制NK细胞活化受体如NKG2D、NKp6、NKp44和NKp30的表达^[70]^。尽管CAFs对于NK细胞的作用已经做了很多研究，但是对于两者的相互作用以及NK细胞反向对于CAFs的调节机制，还有待于进一步发现。

## 7 总结与展望

CAFs和肿瘤浸润性免疫细胞是微环境中关键的组成成分，二者对于肿瘤的发生发展起到了不可或缺的调控作用。在本文中，我们着重讨论了CAFs对于TILs、TAMs、DCs、MDSCs和NKs的调控作用，并对未来可能的研究方向和治疗靶点进行了展望。CAFs可以分泌多种细胞因子以及通过多种通路实现对于肿瘤细胞、免疫细胞的调控。三者之间，形成了复杂的信息网络，共同促进肿瘤的发生发展，尽管相关的研究越来越多，但是三者之间的复杂关系仍然等待着进一步的揭示。近年来，有人通过使用纳米颗粒增强计算机扫描成像的方法，来探究微环境中免疫细胞的变化，以此评估免疫治疗的效果^[71]^。受此启发，考虑是否未来可以采用类似的方法来研究微环境中三者的作用关系。在治疗方面，由于通路很多，如何选择一个合适的通路来保证治疗的有效性，是一个值得考虑的方向。此外，CAFs是一个异质性群体，不同的CAFs亚群在肿瘤的免疫调控方面也有着不同的作用，如何区分不同亚型以及其各自的免疫调控机制，也期待着我们进一步的研究。
